# Cord cross-sectional area at foramen magnum as a correlate of disability in amyotrophic lateral sclerosis

**DOI:** 10.1186/s41747-018-0045-6

**Published:** 2018-06-22

**Authors:** Niccolò Piaggio, Matteo Pardini, Luca Roccatagliata, Carlo Scialò, Corrado Cabona, Laura Bonzano, Matilde Inglese, Giovanni L. Mancardi, Claudia Caponnetto

**Affiliations:** 1 Department of Neuroscience, Rehabilitation, Ophthalmology, Genetics, Maternal and Child Health, University of Genova and IRCCS Azienda Ospedale Università San Martino-IST, Largo Rosanna Benzi 10, 16132 Genoa, Italy; 2 Department of Health Sciences (DISSAL), University of Genova, Via Pastore 1, 16132 Genoa, Italy; 3 Department of Diagnostic and Interventional Neuroradiology, IRCCS AOU San Martino – IST, Largo Rosanna Benzi 10, 16132 Genoa, Italy; 4 Department of Neuroscience, Rehabilitation, Ophthalmology, Genetics, Maternal and Child Health, University of Genova and IRCCS Azienda Ospedale Università San Martino-IST; International School for Advanced Studies (SISSA), Via Bonomea 265, Trieste, Italy; 5 Department of Neurology, Icahn School of Medicine at Mount Sinai, New York, NY USA; 6 Department of Radiology, Icahn School of Medicine at Mount Sinai, New York, NY USA; 7 Department of Neuroscience, Icahn School of Medicine at Mount Sinai, New York, NY USA; 8 IRCCS Azienda Ospedale Università San Martino-IST, Largo Rosanna Benzi 10, 16132 Genoa, Italy

**Keywords:** Amyotrophic lateral sclerosis (ALS), Cervical cord atrophy, Foramen magnum, Magnetic resonance imaging (MRI)

## Abstract

Spinal cord atrophy is one of the hallmarks of amyotrophic lateral sclerosis (ALS); however, it is not routinely assessed in routine clinical practice. In the present study, we evaluated whether spinal cord cross-sectional area measured at the foramen magnum level using a magnetic resonance imaging head scan represents a clinically meaningful measure to be added to the whole-brain volume assessment. Using an active surface approach, we measured the cord area at the foramen magnum and brain parenchymal fraction on T1-weighted three-dimensional spoiled gradient recalled head scans in two groups of subjects: 23 patients with ALS (males/females, 13/10; mean ± standard deviation [SD] age 61.7 ± 10.3 years; median ALS Functional Rating Scale–Revised score 39, range 27–46) and 18 age- and sex-matched healthy volunteers (mean ± SD age 55.7 ± 10.2 years). Spinal cord area at the foramen magnum was significantly less in patients than in control subjects and was significantly correlated with disability as measured with the ALS Functional Rating Scale–Revised (ρ = 0.593, *p* <  0.005). This correlation remained significant after taking into account inter-individual differences in brain parenchymal fraction (ρ = 0.684, *p* <  0.001). Our data show that spinal cord area at the foramen magnum correlates with disability in ALS independently of whole-brain atrophy, thus indicating its potential as a disease biomarker.

## Key points

- Both brain and cervical spinal cord atrophy can be evaluated on the basis of a single three-dimensional T1-weighted acquisition.
- Spinal cord cross-sectional area at the foramen magnum is reduced in patients with ALS compared with healthy control subjects.
- Spinal cord cross-sectional area reduction at the foramen magnum correlates with clinical severity scores in ALS.

## Background

Amyotrophic lateral sclerosis (ALS) is a degenerative disease involving both upper and lower motor neurons and ultimately leads to a fatal outcome in a variably short period of time (median survival 3 years). After an insidious and non-specific onset, the patient experiences progressively worsening functional deficits, such as weakness; fatigue; muscle atrophy; and, as the diseases progresses, loss of control of respiratory muscles, which is the major cause of mortality [1]. Several neuroprotective agents and neuroregenerative approaches are currently under study, and their efficacy is being assessed with functional tests, clinical scales and survival rates.

Magnetic resonance imaging (MRI) of both brain and spinal cord in ALS [2] offers potentially valuable non-invasive radiological surrogate biomarkers at levels of both the whole brain and the region-of-interest [3]. However, even though the spinal cord is affected by ALS and is always assessed for diagnostic purposes, it is rarely investigated in research studies [3, 4]. Despite this, different studies showed a significant relationship of disability with MRI metrics of spinal cord damage, such as diffusion MRI [2, 5], and with cervical cord volume loss, usually assessed at the C2-C3 level [6].

A recent study proposed to measure the spinal cord cross-sectional area at the level of the foramen magnum (foramen magnum cord area [FMCA]) instead of at the usual C2-C3 level and validated it as a surrogate marker in multiple sclerosis [7]. A possible advantage of this approach is the possibility of obtaining a relatively reliable spinal cord atrophy index from a structural brain scan without the need to perform a high-resolution cervical cord acquisition. In fact, even though in most standard brain three-dimensional T1 scans the C2-C3 level is inconsistently included, the foramen magnum region, containing the gradual junction between the caudal medulla oblongata and the rostral cervical cord (to simplify, hereafter referred as *cord*), is generally always included. Therefore, the aims of our present study were (1) to apply FMCA measures in patients with ALS and test whether this measure is independent of whole-brain atrophy, (2) to assess the potential correlation between FMCA and cord area measured at the C2-C3 level (C2-C3 area), and (3) to investigate the clinical relevance of FMCA measures by assessing its relationship with clinical disability.

## Methods

### Subjects

Twenty-three subjects (males/females = 13/10; mean ± standard deviation [SD] age 61.7 ± 10.3 years) diagnosed with probable or definite ALS according to the El Escorial revised criteria [8] were enrolled in this study. All patients were clinically evaluated with the ALS Functional Rating Scale–Revised (ALSFRS-R) [9], always by the same experienced neurologist, who had 8 years of experience. Eighteen healthy age- and sex-matched subjects (mean ± SD age 55.7 ± 10.2 years) not related to the enrolled patients were also enrolled specifically for this study as a healthy control group of volunteers. All study procedures were approved by our institution’s ethics committee and conducted according to the Declaration of Helsinki. Informed consent to participate and for publication of the results was acquired from all participants.

### MRI protocol

Within 5 days of clinical evaluation, all subjects underwent a routine clinical MRI protocol, including a standardized T1-weighted brain volumetric scan acquired with a 1.5-T system (SIGNA; General Electric Healthcare, Milwaukee, WI, USA) using an eight-channel high-resolution brain array coil. The sequence obtained was a sagittal three-dimensional spoiled gradient recalled sequence with the following technical parameters: repetition time 28 ms, echo time 6 ms, flip angle 30 degrees, field of view 256 × 256 mm, in-plane matrix 256 × 256, slice thickness 1.3 mm. Patients were standardly positioned with particular care to avoid neck hyperextension, which is described to cause artifacts and unusual inflections of the cord [10, 11]. Foam pads were placed around the patient’s head to increase comfort and reduce motion artifacts. All scanned volumes included the foramen magnum area.

### Image analysis

**Fig. 1:**
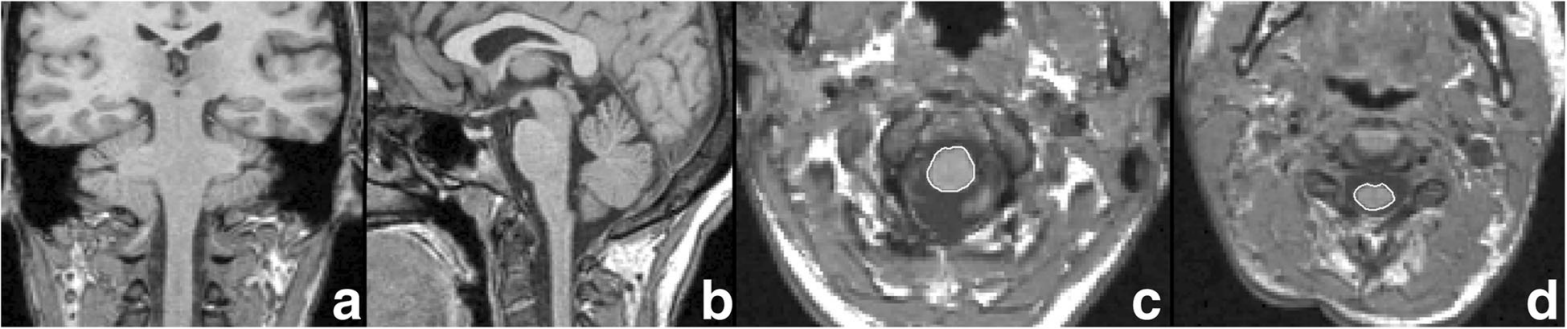
**a** and **b** Three-dimensional T1-weighted images volume-realigned perpendicularly to the main axis of the cord. Re-oriented axial views at the level of the foramen magnum (**c**) and at the C2-C3 level (**d**) depict the medians of five regions of interest for the quantification of cord area

First, all images were visually checked for quality before analysis. An experienced neuroradiologist with more than 5 years of experience, who was blinded to group assignment and to the clinical ALSFRS-R results, performed the image post-processing as previously described [7] using Jim software (version 6; Xinapse Systems, West Bergholt, UK). Briefly, multi-planar reconstruction of the volumetric T1-weighted scan was re-oriented on an axial plane in order to obtain a cross-section of the cord perpendicularly to its long axis (Fig. 1a, b). A 1.0 × 1.0 × 1.3-mm slice was then visually identified to isolate the foramen magnum landmark. At this level, the centre of the cord was manually marked with a seed point, and the Cord Finder tool (available in Jim Software) was run to automatically contour the cord parenchyma (Fig. 1c) using an active surface algorithm [12]. Each segmentation result underwent an accuracy check and, if necessary, manual editing. To enhance accuracy, cord area was assessed via region-of-interest statistics in the Jim environment on five different contiguous 1.3-mm-thick parallel slices for each patient starting from the slice at the foramen magnum and extending downwards. Moreover, we evaluated on which patients the scan field of view covered the C2-C3 cord region satisfactorily, and subsequently we calculated the cord area at that level (C2-C3 area) using the same procedure applied for FMCA. From the volume obtained with the same sequence, brain parenchymal fraction (BPF), defined by the ratio between brain parenchymal (the sum of white and grey matter) and intracranial (the sum of white and grey matter and cerebrospinal fluid) volumes, was estimated for all subjects using the Statistical Parametric Mapping 12 spm_segment pipeline [13].

### Statistical analysis

All statistical analyses were carried out using IBM SPSS Statistics version 20 software (IBM, Armonk, NY, USA). FMCA, C2-C3 area and BPF samples were tested for normality with the Shapiro-Wilk test. An independent samples *t* test between the ALS and control groups was performed to assess if there were significant differences in age, BPF and FMCA mean values. Pearson’s correlations were then used to assess the association between FMCA and ALSFRS-R and between FMCA and C2-C3 area. A partial correlation test controlling for BPF values was performed to test if the FMCA relationship to ALSFRS-R was independent from inter-individual BPF variabilities. A further partial correlation test taking age into account was performed to verify the impact of age on the FMCA/ALSFRS-R correlation. Data are reported as mean ± SD.

## Results

### Clinical evaluation

In our sample, the age of patients and disease duration were, respectively, 61.7 ± 10.3 years and 9.4 ± 6.4 months. Clinical evaluation of our group of patients with ALS resulted in a median ALSFRS-R score of 39, with a range from 27 to 46.

### Differences in MRI metrics between patients and control subjects

In 23 subjects (14 patients and 9 control subjects), the C2-C3 cord region was consistently included in the MRI field of view. Overall, 18 cord contours had to be manually edited. The image processing of each patient required around 15 minutes. FMCA, C2-C3 area, and BPF values appeared normally distributed in the patients with ALS and the healthy control subjects.

**Fig. 2:**
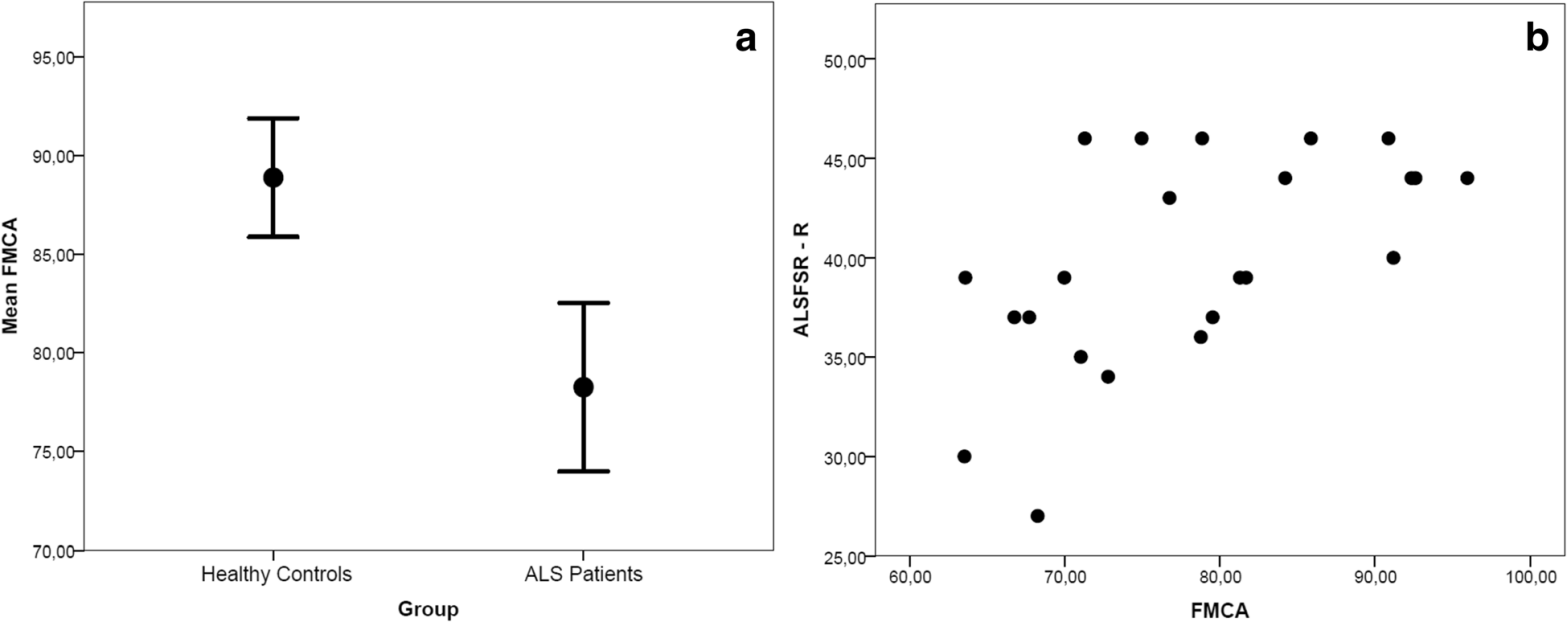
**a** Graph depicting significant difference in the foramen magnum cord area (FMCA) (mm^2^) between the two groups with 95% CIs. **b** Plot showing the correlation between FMCA (mm^2^) and Functional Rating Scale–Revised (ALSFRS-R) values of patients with amyotrophic lateral sclerosis (ALS)

The independent samples *t* test between patients with ALS and healthy control subjects resulted in a significant difference in FMCA values: 78.3 ± 9.9 mm^2^ and 88.9 ± SD 6.0 mm^2^, respectively (*p* < 0.001) (Fig. 2a). Conversely, age and BPF values did not differ significantly between the patients with ALS and healthy control subjects: 61.7 ± 10.25 years and 55.7 ± 10.2 years, respectively (*p* = 0.732), and 0.78 ± 0.06 and 0.79 ± 0.06, respectively (*p* = 0.331). In the subsample of 23 subjects in whom C2-C3 area was obtainable, C2-C3 area did not differ significantly between patients and control subjects: 70.27 ± 6.12 mm^2^ and 74.84 ± 4.9 mm^2^, respectively (*p* = 0.077). Because multiple independent samples *t* tests were performed, the Bonferroni correction was applied (with a threshold of *p* = 0.00125); thus, the difference in FMCA values remained significant at *p* = 0.005.

### Correlation between clinical and MRI measures

The correlation between FMCA and C2-C3 area (in the subset of 23 subjects in whom this value was obtainable) was significant (Pearson’s ρ = 0.778, *p* < 0.001). The correlation between ALSFRS-R and FMCA values was significant (Pearson’s ρ = 0.593, *p* < 0.005), with subjects presenting with a more severe ALS clinical disability score having a smaller FMCA area (Fig. 2b); this correlation remained significant even after taking into account possible biasing variables such as age (ρ = 0.550, *p* = 0.008) and BPF (ρ = 0.684, *p* < 0.001). Moreover, the correlation between ALSFRS-R and C2-C3 area (in the 14 patients where applicable) also was significant (Pearson’s ρ = 0.556, *p* = 0.039). Results are summarized in Table 1.

**Table 1:** Demographics and magnetic resonance imaging results

	Patients with ALS	Healthy control subjects	*p* Value
Subjects, total (males/females)	23 (13/10)	18 (5/13)	–
Age, years, mean ± SD	61.7 ± 10.3	55.7 ± 10.2	0.738
FMCA, mm^2^, mean ± SD	78.3 ± 9.9	88.9 ± 6.0	< 0.005^a^
BPF, mean ± SD	0.78 ± 0.06	0.79 ± 0.06	0.336
ALSFRS-R, median; range	39; 27–46	–	–
Disease duration, months, mean ± SD	9.43 ± 6.41	–	–

*Abbreviations: ALS* Amyotrophic lateral sclerosis; *FMCA* Foramen magnum cord area; *BPF* Brain parenchymal fraction; *ALSFRS-R* ALS Functional Rating Scale–Revised

^a^ Corrected for multiple comparisons with Bonferroni post hoc test

## Discussion

Overall, our findings show that FMCA as well as C2-C3 cord area correlates with clinical disability and that the correlation between FMCA and clinical disability score is independent of brain atrophy. Furthermore, our data show that our metric (FMCA) could be assessed with clinical MRI data more easily than C2-C3 cord area, which was satisfactorily included only in a limited number of scans (54%).

Furthermore, though the C2-C3 cord area differed between patients and control subjects, this difference did not reach statistical significance, probably due to the small number of subjects for whom the C2-C3 area was available compared with the whole study population. Although atrophy in the cervical cord segment is a key pathological element of ALS, its optimal visual quantification is difficult in clinical practice; therefore, the availability of an objective parameter to assess spinal cord volume loss could be very helpful in this context.

To date, in ALS clinical trials, disease severity and progression have usually been evaluated with clinical scores and functional tests. The use of these endpoints typically necessitates long-term assessment of a large number of patients, consequently increasing the cost of clinical trials and potentially discouraging the evaluation of novel treatments, particularly neuroprotective drugs. Sensitive imaging biomarkers can be useful to this end, such as by reducing sample size in pilot studies. In ALS, given the involvement of both the brain and the spinal cord, a lengthy brain-spinal cord protocol is needed to evaluate these two regions using available radiological surrogate markers. Obtaining cord atrophy information from a fast “single anatomical district” protocol is particularly advantageous because it spares the necessity of a dedicated spinal cord acquisition, which would prolong the scan time, consequently challenging patients with ALS. Because FMCA can be computed using conventional volumetric head scans, its evaluation could represent a high-yield addition to clinical and research imaging protocols to systematically, albeit indirectly, assess spinal cord volume loss in ALS. Of note, the computation of FMCA is equally feasible using previously acquired datasets, which involves the interesting potential to retrospectively review a large number of clinical scans.

In this study, we measured FMCA, a straightforward MRI indicator that requires very light processing time and basic skills, and we investigated its relationship to the current clinical scale. Our results are consistent with literature findings that assess cord atrophy at levels different from the foramen magnum and correlate it with disability clinical scores in ALS [6].

The independence of FMCA correlation with the clinical marker ALSFRS-R from BPF values confirms the additional information that this novel measure could provide in a clinical MRI analysis protocol, suggesting that a combined imaging biomarker could further improve accuracy in disease monitoring [14] because two different and complementary sets of information can be acquired from a single head scan. The advantages in terms of improved robustness of a brain-cervical composite atrophy marker compared with the single measures are already validated in the literature for other neurological conditions [15].

A limitation of our study is that it was conducted with a relatively small number of subjects, partly owing to the moderate prevalence of the disease and partly to the tolerability of MRI acquisitions of patients with ALS; hence, further analysis of a larger cohort of patients followed over time is desirable. We acknowledge that primary motor cortex atrophy could represent a more precise measure of upper motor neuron degeneration than BPF [16], and thus future studies are warranted to explore the relationship between FMCA and primary motor cortex thickness in ALS. Despite this, given the focus of the present work on metrics that could easily be used in clinical practice, we decided to use BPF as a proxy marker of upper motor neuron pathology because, to date, thickness-based measures have not been widely used outside the clinical setting.

We show that FMCA is associated with disability independently of BPF. Despite this, we acknowledge that FMCA (but also C2-C3 area) does not represent a pure measure of lower motor neuron pathology, given the impact of pyramidal tract volume loss on cord measures. Future studies whose aim is to compare FMCA with cervical grey matter area are warranted to tease out the relative role of upper and lower motor neuron pathology in FMCA changes. We also acknowledge that FMCA cannot be considered a pure measure of upper and lower motor neuron degeneration; however, our data show that FMCA conveys information different from BPF (which is widely used as a proxy of upper motor neuron degeneration in ALS [17]) and it is more easily quantifiable in clinical imaging than C2-C3 area.

In conclusion, our data show that FMCA correlates with disability in patients with ALS independently of whole-brain atrophy, thus suggesting that it could be considered as a disease biomarker with the potential to become a surrogate endpoint in research trials, possibly alongside other clinical and imaging tools, in order to create composite indicators of damage.

## Abbreviations

ALS: Amyotrophic lateral sclerosis
ALSFRS-R: ALS Functional Rating Scale–Revised
BPF: Brain parenchymal fraction
FMCA: Foramen magnum cord area
MRI: Magnetic resonance imaging
SD: Standard deviation.
